# Genes and genetics belong to maize haploid induction

**DOI:** 10.3389/fpls.2025.1634053

**Published:** 2025-08-05

**Authors:** Kanogporn Khammona, Abil Dermail, Yu-Ru Chen, Thomas Lübberstedt, Samart Wanchana, Theerayut Toojinda, Siwaret Arikit, Vinitchan Ruanjaichon

**Affiliations:** ^1^ Department of Agronomy, Faculty of Agriculture at Kamphaeng Saen, Kasetsart University, Nakhon Pathom, Thailand; ^2^ National Center for Genetic Engineering and Biotechnology (BIOTEC) , Khlong Nueng, Thailand; ^3^ Department of Agronomy, Faculty of Agriculture, Khon Kaen University, Khon Kaen, Thailand; ^4^ Department of Agronomy, Iowa State University, Ames, IA, United States; ^5^ Rice Science Center, Kasetsart University, Nakhon Pathom, Thailand

**Keywords:** paternal haploid inducer, maternal haploid inducer, R1-nj, chromosome doubling, spontaneous chromosome genome doubling

## Abstract

Maize (*Zea mays* L.) is a globally significant cereal crop with diverse food, feed, and industry uses. The rapid development of homozygous inbred lines via double haploid (DH) technology has revolutionized maize breeding, reducing the time and cost required for cultivar improvement. This review synthesizes advances in haploid induction systems, focusing on the genetic mechanisms underlying both paternal and maternal inducers. Key genes such as *IG1, MTL/ZmPLA1/NLD, ZmDMP, ZmPLD3, ZmPOD65*, and the centromeric histone variant *CENH3* are examined for their roles in haploid embryo formation. Methods of haploid identification based on DNA content and phenotypic markers (e.g., *R1-navajo* and *Pl1* genes) are critically assessed, including recent innovations that enhance selection accuracy. Additionally, the integration of kernel oil content (KOC) as a quantitative trait for haploid discrimination is discussed. Progress in artificial and spontaneous chromosome doubling techniques, particularly the roles of colchicine, N_2_O treatments, and identified QTL governing spontaneous haploid genome doubling (SHGD), are highlighted. This review underscores the transformative potential of combining novel genetic tools, precision phenotyping, and genome-editing strategies to further optimize DH technology for maize improvement, ultimately facilitating next-generation plant breeding programs.

## 1 Introduction

Maize (*Zea mays* L.) domesticated in southern Mexico/Mesoamerica over 9,000 years ago (Awika, 2011; Erenstein et al., 2022; Kennett et al., 2020), is one of the most important annual cereal crops worldwide (Rouf-Shah et al., 2016). Current global production exceeds 1,000 tons (García-Lara and Serna-Saldivar, 2019), with cultivation spanning approximately 197 million hectares. This area includes significant regions in Sub-Saharan Africa (SSA), Asia, and Latin America, with the Americas contributing about 50% of total production, followed by Asia (32%), Europe (11%), and Africa (7.4%) (Erenstein et al., 2022; FAOStat, 2021). Maize is distinguished by its broad phenotypic and functional diversity, often classified by kernel color (e.g., yellow, white, blue) and end-use types such as sweet corn, waxy corn, baby corn, dent/flint, popcorn, high-amylose, high-oil, and high-protein corn (Serna-Saldivar and Perez Carrillo, 2019). Beyond its agronomic and economic significance, maize serves as a vital source of human nutrition. Its contribution to dietary energy and protein intake is substantial, particularly in regions where it forms a staple component of food systems (Shiferaw et al., 2011).

The development of fully homozygous inbred lines is an important component of maize breeding programs, serving as the foundation for producing parental lines used in hybrid and synthetic variety development (Chaikam et al., 2019a). Throughout the twentieth century, the development of inbred lines primarily relied on six to eight generations of recurrent self-pollination and phenotypic selection to attain the desired level of homozygosity (Hallauer et al., 2010). Over the past two to three decades, however, doubled haploid (DH) technology has emerged as a powerful and time-efficient alternative to the traditional methods. By significantly accelerating the development process and reducing associated costs, DH technology has transformed inbred line production. Two main haploid induction systems have been established within this framework: the paternal haploid inducer and the maternal haploid inducer systems.

Haploid inducers are specialized genetic stocks that, when crossed with a diploid (normal) maize plant, produce ears containing a mixture of diploid (2n) and haploid (n) kernels. This phenomenon, resulting from abnormal fertilization, was first described by Stadler and Randolph in 1929 (Randolph, 1932). Kernels with a haploid embryo typically possess a triploid (3n) endosperm, allowing them to exhibit germination rates comparable to those of kernels with diploid embryos (Coe and Sarkar, 1964; Prasanna et al., 2012). These haploid embryos can subsequently be used to generate DH lines through genome doubling technologies.

Traditional paternal haploid inducers carry the *indeterminate gametophyte (ig)* gene and are employed as the female parent. This variant is considered recessive because homozygous *ig* plants exhibit male sterility in the W23 inbred background, where the mutation originally arose. Heterozygous *Ig/ig* plants segregate in a 1:1 ratio for indeterminate female gametophytes (Kermicle, 1994). However, this approach is less favorable because the nuclear genome of the paternal donor is transmitted to the resulting haploid embryos, which may be undesirable (Trentin et al., 2020). Recent studies reported that the *CENTROMERIC HISTONE3 (CENH3)* gene can induce the formation of androgenic haploids—haploids that retain the male genome while eliminating the female genome. When used on the female side, *CENH3* modification produced approximately 30% androgenic haploids, compared to about 3.6% when used on the male side (Zhao et al., 2013; Kelliher et al., 2016) (**Table 1**).

**Table 1:** Summary of genes related to haploid induction in maize.

Gene	Haploid induction type	Maize variety	Modification technique	HIR (%)	References
*ig*	*Paternal*	W23	Spontaneous	~3	Kermicle (1969)
	*Paternal*	ND	Spontaneous	2.36	Kyada et al. (2024)
*CENH3*	*Paternal*	UFMu-01386	*+/cenh3*	5.2	Wang et al. (2021)
	*Maternal*	UFMu-01386	AcGREEN-tailswap-*CENH3*	3.6	Kelliher et al. (2016)
*mtl/zmpla1/nld*	*Maternal*	Stock 6	Spontaneous	2	Coe (1959)
	*Maternal*	PK6	Spontaneous	6	Barret et al. (2008)
	*Maternal*	CAUHOI	Spontaneous	2	Prigge et al. (2012)
	*Maternal*	A188	GMO	0.5-3.59	Gilles et al. (2017)
	*Maternal*	non-inducer NP2222	transcription-activator-like effector nucleases (TALEN)	6.7	Kelliher et al. (2017)
	*Maternal*	ND	CRISPR/Cas9-mediated knockout	3.7-6.67	Liu et al. (2017)
*zmdmp*	*Maternal*	ND	CRISPR/Cas9-mediated knockout	0.1–0.3	Zhong et al. (2019)
*zmpld3*	*Maternal*	LH244	CRISPR/Cas9-mediated knockout	0.85–0.96	Li et al. (2021)
*zmpod65*	*Maternal*	ND	CRISPR/Cas9-mediated knockout	1–7.7	Jiang et al. (2022)
Gene combination mutations					
*CENH3 + mtl/zmpla1/nld*	*Maternal*	CAUHOI	maize H3.2 N-terminal-tailswap-RFP*-CENH3*	8.8	Meng et al. (2022)
*CENH3 + mtl/zmpla1/nld + zmdmp*	*Maternal*	CAU5	maize H3.2 N-terminal-tailswap-RFP-*CENH3*	16.3	Meng et al. (2022)
*mtl/zmpla1/nld + zmdmp*	*Maternal*	UH400	Spontaneous	8	Prigge et al. (2012)
*mtl/zmpla1/nld + zmdmp*	*Maternal*	CAU5	Spontaneous	~10%	Zhong et al. (2019)
*mtl/zmpla1/nld + zmpld3(+/–) + zmdmp*	*Maternal*	LH244	CRISPR/Cas9-mediated knockout	~7%	Li et al. (2021)
*mtl/zmpla1/nld + zmpld3*	*Maternal*	LH244	CRISPR/Cas9-mediated knockout	~4%	Li et al. (2021)
*mtl/zmpla1/nld + zmdmp*	*Maternal*	LH244	CRISPR/Cas9-mediated knockout	~4-5%	Li et al. (2021)
*zmpld3 + zmdmp*	*Maternal*	LH244	CRISPR/Cas9-mediated knockout	~1%	Li et al. (2021)

*ND, Not Detected.

Maternal haploid inducers commonly contain key genes such as *MATRILINEAL (MTL)/NOT LIKE DAD (NLD)/PHOSPHOLIPASE-A (PLA)* (Liu et al., 2017; Gilles et al., 2017: Kelliher et al., 2017), *DOMAIN OF UNKNOWN FUNCTION 679 MEMBRANE PROTEIN* (*ZmDMP*), *Zea mays PHOSPHOLIPASE D3* (*ZmPLD3*), and *ZmPOD65* gene (**Table 1**). These genes are utilized in the pollen-source parent during haploid induction. The first report of haploid plants in maize was presented simultaneously by Randolph and Stadler at the 1929 meeting of the American Association for the Advancement of Science (Randolph, 1932). Later, in Chase 1947, reported the natural occurrence of haploid plants in commercial inbred lines at a frequency of less than 0.1%. In 1959, Coe reported a haploid induction rate (HIR) of 3.2% in the self-pollinated progeny of inbred line Stock 6, which subsequently became a primary source of germplasm for developing new haploid inducers (Hu et al., 2016). Researchers quickly recognized the breeding potential of these haploid plants (Chase, 1947, 1949), as only the maternal genome is transmitted to the haploid embryos, facilitating efficient selection.

This manuscript will discuss the various methods of haploid detection based on both DNA content and morphological traits, as well as genes involved in haploid induction, along with the associated challenges and opportunities in this field. Therefore, the aim of this manuscript was to (1) explain their genetic basis of the genes belonging to haploid inducers and their role in haploid induction, and (2) review novel insights the spontaneous haploid genome doubling (SHGD) in maize.

## 2 The genetic mechanisms behind haploid induction

### 2.1 Genes involved in paternal haploid induction

#### 2.1.1 *IG1* gene

In maize, the *Indeterminate gametophyte1* (*ig1*) mutation was first observed as a spontaneous mutation in the inbred line Wisconsin-23 (W23), resulted in approximately 3% HIR (Kermicle, 1969). The *ig1* gene is localized on the long arm of chromosome 3, 90 cM from the most distal locus on the short arm (Kermicle and Demopulos-Rodriguez, 1980; Coe, 1992). It encodes a *LATERAL ORGAN BOUNDARIES (LOB)-domain protein* (Evans, 2007), which is associated with a large, plant-specific family of transcription factors (Husbands et al., 2007). This single recessive gene causes a range of embryological abnormalities, including atypical fertilization events that lead to ploidy variation in both the embryo and the endosperm (Kermicle, 1969, 1971, 1994; Lin, 1981).

Several embryological abnormalities have been described in *ig1* embryo sacs, both in mature (Lin, 1978) and developing stages (Lin, 1981). Lin (1978) observed the phenotype of maize ovules from the Wisconsin dent inbred line W23 (*ig1/ig1*) when used as the female parent at the time of silk emergence, corresponding to the mature female gametophyte stage. The results revealed that the structure of mature embryo sacs in *ig1/ig1* plants differs markedly from that in wild-type (*Ig1/Ig1*) plants in several respects: (1) Wild-type plants consistently produced a single micropylar cell (egg), whereas *ig1* embryo sacs exhibited a range of 0 to 0.5 structurally analogous cells per sac. (2) In *ig1* embryo sacs, the primary central cell was frequently accompanied by one or two additional, smaller central cells. These additional cells were occasionally binucleate but more often uninucleate. In contrast, wild-type embryo sacs invariably contained a single central cell. (3) The primary central cell in *ig1* sacs exhibited considerable variability, containing between one and six polar nuclei. Wild-type sacs uniformly harbored two polar nuclei. (4) In *ig1* embryo sacs, some polar nuclei were dispersed throughout the cell, while others occupied the typical apical position near the egg cell, opposite the micropyle. During double fertilization, only a subset of the micropylar and polar nuclei appeared to be functionally competent. The average number of unfertilized micropylar nuclei was 0.88 per ovule, while unfertilized polar nuclei averaged 0.75 per ovule. These unfertilized nuclei included both those located in accessory central cells and those in the primary central cell, but were displaced from their typical spatial location.

Lin (1981) analyzed 62 mature *ig1* female gametophytes from the maize inbred line W23. Of these, only two exhibited the defining characteristics of the wild-type structure: a single micropylar cell —consistent with synergid regression in W23, leaving only the egg cell—and a binucleate central cell with nuclei positioned adjacent to the egg. In the *ig1* samples, the number of cells present in the micropylar region ranged from zero to five, averaging 1.96. The number of polar nuclei averaged 3.42 per gametophyte, and approximately 18% of the embryo sacs contained one or more accessory central cells. Notably, among the ten cases examined, fertilization events involving accessory central cells were not observed. Correspondingly, the phenomenon of endosperm doubleness was absent in mature seeds. Further cytological analysis by Lin revealed that in kernels developing on *ig1/ig1* plants, the number of maternally derived chromosome sets in the endosperm corresponded to the number of polar nuclei closely appressed to the egg cell, rather than with the total number of polar nuclei present in the primary central cell (Kermicle, 1994).

Huang and Sheridan (1996) conducted a comparative study of nuclear dynamics and microtubule organization in wild-type plants and *ig1* mutants. In *ig1* mutants, the second and third mitotic divisions within the embryo sacs are not fully synchronized. In addition, supernumerary mitoses may occur, either throughout all nuclei or specifically in the micropylar or central regions. Following cellularization, individual micropylar cells can undergo mitosis. Aberrant microtubule behavior in the mutant leads to irregular nuclear positioning, asynchronous microtubule organization among nuclear pairs, and abnormal phragmoplast formation after the third mitotic event. These results indicate that the ig1 gene plays a primary role in the regulation of nuclear division and a secondary, yet significant, role in modulating microtubule dynamics. The gene’s influence on the cytoskeleton likely contributes to proper polarization and nuclear migration, processes that are critical for normal embryo sac development and cellular differentiation (Huang and Sheridan, 1996).

Evans (2007) positionally cloned *ig1* in a genetic cross between *ig1*-Ow23/+Mo17 females and +/+Mo17 males. Fine mapping using simple sequence repeat (SSR) markers localized *ig1* between umc1311 and umc1973 on maize chromosome 3. The gene was subsequently cloned based on the genome sequence of the orthologous region in rice. A second *ig1* mutant allele was identified via a non-complementation screen involving active Mutator transposable element lines. Homozygous *ig1* mutants exhibit pronounced developmental abnormalities, including altered leaf morphology and defective embryo sac formation. Mutant leaves display disrupted abaxial–adaxial polarity and fail to suppress the expression of meristem-specific knotted-like homeobox (knox) genes in developing leaf primordia. This mis- regulation results in the persistence of proliferative, stem cell-like identity. Notably, despite phenotypic similarities between *ig1-O* leaves and embryo sacs, ectopic knox gene expression is not detected in *ig1-O* embryo sacs (Evans, 2007).

Recently, Kyada et al. (2024) identified eight distinct haplotypes of the *Ig1* gene across eight wild-type inbred lines (with *Ig1* comprising two exons) and two mutant inbreds (*ig1*, with a single exon). The study reported a haplotype diversity index of 0.956 and a nucleotide diversity (Pi) value of 0.013. The insertion of Hopscotch retrotransposon in *ig1* resulted in a truncated protein with 138 amino acids, in contrast to 260 amino acids in wild-type *IG1* protein. The study also revealed that the disruption of the leucine zipper domain in the mutant protein accounted for its loss of function. To facilitate genotyping, a functional marker, MGU-IG1-Hopscotch, specific to the Hopscotch insertion, was developed and successfully validated in five F_2_ populations. Across these populations, the average HIR was notably higher in mutant homozygotes (*ig1/ig1*; 2.36%) than in heterozygotes (*Ig1/ig1*, 1.49%). Furthermore, *ig1*-positive plants exhibited a twin-embryo seed phenotype at frequencies ranging from 0 to 1.6%.

### 2.2 Genes involved in maternal haploid induction

#### 2.2.1 *MTL/ZmPLA1/NLD* gene

Since 1959, Coe observed self-pollinated progenies of an inbred line, designated Stock 6, produced 343 haploids out of 10,616 plants, corresponding to an average haploid induction frequency of 2%, with a maximum of 3.23% when used as a male parent. Subsequently, Deimling et al. (1997) identified a major QTL on chromosome 1 (interval 1.03–1.06) from a cross between W23ig and Stock6. Later, Barret et al. (2008) reported the same chromosomal region (bin 1.04) in a study of 471 F_2_ plants derived from a cross between DH99, a non-inducer female parent, and PK6, a non-glossy maize line developed from Stock6 with an average HIR of 6%. Genotyping of the 19 highest-inducing F_2_ plants using 101 microsatellite markers led to the identification of gynogenesis inducer 1 (ggi1) (Barret et al., 2008).

Prigge et al. (2012) evaluated HIR across 1,061 progenies derived from four segregating populations involving two temperate haploid inducers UH400 (HIR = 8%) and CAUHOI (HIR = 2%), alongside one temperate and two tropical non-inducer inbreds (HIR = 0%). Each population was examined over three successive generations. The QTL analysis identified *qhir1* as a major locus, explaining up to 66% of the observed genetic variance in the three populations that involved a non-inducer parent. The allele enhancing HIR at this locus was contributed by UH400.

Dong et al. (2013) conducted fine-mapping of the *qhir1* locus and identified markers closely linked to this region. Through fine-mapping efforts involving 14,375 F_2_ individuals derived from a cross between 1680 and UH400, polymorphic markers specific to the *qhir1* region were developed. This work successfully narrowed the original *qhir1* interval from 3.57Mb to a 243-kb region flanked by markers X291 and X263. Subsequently, Hu et al. (2016) employed genome-wide association studies (GWAS) and further dissected the *qhir1* locus into two sub-QTLs: *qhir11* (0.54 Mb), which contained the 243-kb interval previously identified by Dong et al. (2013), and *qhir12* (3.97 Mb), within the broader 50.34 Mb *qhir1* region. Then, Nair et al. (2017) characterized the functional contributions of these sub-regions through a cross between CML269 (a non-inducer) and TAIL8 (a tropicalized haploid inducer). Their analysis revealed that only the *qhir11* sub-region significantly affected haploid induction ability, and was also associated with traits such as segregation distortion and kernel abortion, hallmark features linked to maternal haploid induction.

In 2017, the *ZmPLA1/MTL/NLD* characterization was studied and reported by three research groups identified GRMZM2G471240, a gene encoding a patatin-like phospholipase, as a candidate. A 4-bp insertion in its fourth exon induced a frameshift at amino acid 380, resulting in 20 altered residues and a premature stop codon that truncated the protein by 29 amino acids. Multiple functional validations were set through RNA interference (RNAi), transcription-activator-like effector nucleases (TALEN), and genome editing to confirm the role of this gene in haploid induction (Kelliher et al., 2017; Liu et al., 2017; Gilles et al., 2017).

Later, Gilles et al. (2021) observed and characterized *NLD* localization in maize sperm cells and revealed that the gene is localized on the endo-plasma membrane of sperm cells and is responsible for sperm cells’ PI(4,5)P2 signaling to the embryo sac and helps to deliver the sperm cells. Therefore, they were suggested that the loss of function of *NLD* would impact sperm cells’ failure to localize in the female egg, leading to the formation of haploid embryos with only the chromosomes from the female parent.

Most recently, Jiang et al. (2022) conducted a comprehensive multi-omics analysis integrating transcriptomic, proteomic, metabolomic, and protein modification datasets to explore *zmpla1* mutant anthers. Their findings indicated that differential molecular entities were significantly enriched in pathways associated with the oxidative stress response. Notably, they discovered that inducing a reactive oxygen species (ROS) burst via chemical treatment of pollen could trigger haploid induction, suggesting that ROS-mediated signaling plays a critical regulatory role in the haploid induction process.

#### 2.2.2 *ZmDMP* gene

Prigge et al. (2012) identified seven QTL across five chromosomes in the CAUHOI × UH400 population, with *qhir8* on chromosome 9 accounting for 20% of the HIR variation across three generations. At the time of discovery, *qhir8* was classified as a minor QTL. Subsequently, Liu et al. (2015) fine-mapped the *qhir8* region and assessed its impact on HIR, segregation distortion (SD), and embryo abortion (EmA). A total of 3,989 F_2_ plants derived from the CAUHOI × UH400 cross were screened for recombinants within the *qhir8* region. Later, F_2_ plants and F_3_ progenies from 34 recombinant lines were evaluated for HIR, SD, and EmA, using 31 markers spanning the target region. Their analysis confirmed that *qhir8* significantly enhanced HIR and EmA, but had no discernible effect on SD. The *qhir8* locus was narrowed down to a 789 kb region flanked by markers 4292232 and umc1867.

Zhong et al. (2019) leveraged a fine-mapping approach using 16 F_3_ families derived from a cross between CAU5 and CAUHOI, narrowing the *qhir8* region to a 138-kb region flanked by markers ZS4307 and ZS4446. Sequencing within this region utilized two positive bacterial artificial chromosome (BAC) clones from CAU5, yielding detailed sequence information and additional polymorphic markers. Using these markers, the mapping interval was further reduced to a 318-bp region through analysis of 21 newly generated F_3_ families. Ultimately, the *qhir8* locus was localized within the coding sequence of *GRMZM2G465053*, which encodes a DUF679 domain-containing membrane protein, subsequently designated *ZmDMP*. A single-nucleotide polymorphism (SNP) was identified within this region: a thymine (T) in CAUHOI was replaced by a cytosine (C) in CAU5, located 131 bp downstream of the start codon (ATG). This SNP resulted in an amino acid substitution from methionine to threonine, implicating this mutation as the causal variant underlying the enhanced HIR observed in CAU5.

Functional validation was performed by generating *ZmDMP* knockout lines (*zmdmp-ko*) using the clustered regularly interspaced short palindromic repeats (CRISPR)–CRISPR-associated protein 9 (Cas9) system. The knockout induced haploid formation with a HIR of 0.1–0.3%, and when combined with *mtl/zmpla1/nld* mutations, the HIR was elevated by 5–6-fold. The haploid status of the progeny was verified using 10 polymorphic molecular markers and flow cytometry, confirming the presence of only the maternal genotype in all cases.

Consistent with the functional profile of *qhir8*, *ZmDMP* disruption also led to an increased frequency of endosperm-aborted kernels (EnAs). Expression analysis using quantitative reverse transcription PCR (qRT–PCR) demonstrated that *ZmDMP* and *zmdmp* are highly expressed in mature pollen, with markedly lower expression levels in immature anthers and kernels at various developmental stages. Notably, expression of *zmdmp* in mature pollen was significantly higher than that of *ZmDMP*, likely reflecting a feedback regulatory mechanism. These findings suggest that *ZmDMP* plays a significant role during the late stages of male gametophyte development and establishes it as a key genetic determinant of haploid induction. The results clearly recognized that the *zmdmp* gene is responsible and plays a critical role in haploid induction in maize (Zhong et al., 2019).

#### 2.2.3 *ZmPLD3* gene

In Li et al., 2021, characterized the role of phospholipase-mediated haploid induction in maize by analyzing RNA-seq data previously reported by Walley et al. (2016), which profiled different tissues of maize inbred line B73. They aimed to identify pollen-specific members of the phospholipase gene family. Among the candidates, only one gene, *ZmPLD3*, was exclusively expressed in pollen and significantly upregulated in the *mtl/zmpla1/nld* mutant background. Quantitative reverse transcription PCR (qRT–PCR) further confirmed that *ZmPLD3* was highly expressed in mature pollen relative to anthers at various developmental stages.

*ZmPLD3* encodes a, putative *phospholipase D* (*PLD*), locate on chromosome 6, characterized by its conserved hydrolytic active-site motif (HKD motif, HxKxxxxD) (Wang, 2001). Sequence analysis revealed the presence of two HKD domains within the *ZmPLD3* protein. To elucidate its functional role, CRISPR-Cas9-mediated gene editing was employed to generate two independent knockout lines, *zmpld3–*1 and *zmpld3*-2, which were subsequently selected for further study. The HIRs of *zmpld3–*1 and *zmpld3–*2 were 0.96% and 0.85%, respectively, which did not significantly differ from that of *mtl/zmpla1/nld* (1.2%).

To assess potential genetic interactions between *ZmPLD3* and previously reported haploid induction genes, double mutants (*zmpld3-mtl, zmpld3-zmdmp*, and *mtl-zmdmp*) were generated through hybridization of the corresponding single mutants. Statistical analyses revealed that the combination of *zmpld3* and *mtl/zmpla1/nld* elevated the HIR to approximately 4%. In contrast, the HIR of the *zmpld3-zmdmp* double mutant was not significantly higher than that of the *zmpld3* single mutant, although *zmpld3* alone exhibited a significantly higher HIR compared to *zmdmp*.

Subcellular localization of *ZmPLD3* was examined using a maize protoplast system co-expressing compartment-specific marker protein. The results indicated that *ZmPLD3* localizes to multiple cellular compartments, including the endoplasmic reticulum, plastids, Golgi apparatus, and cytosol, but is likely absent from the plasma membrane, mitochondria, pre-vacuolar compartment, nucleus, and peroxisomes.

#### 2.2.4 *ZmPOD65* gene

Recently, Jiang et al. (2022) discovered that a simple chemical treatment of pollen with ROS reagents could effectively induce haploid formation in maize. Through an integrated multi-omics analysis—including transcriptomics, metabolomics, quantitative proteomics, and protein modification profiling—of *zmpla1* mutant anthers, they identified three genes encoding sperm-specific peroxidases: *ZmPOD65* (*Zm00001d017996*), *ZmPOD60-1* (*Zm00001d027710*), and *ZmPOD60-2* (*Zm00001d048413*). Targeted mutagenesis of *ZmPOD65* using CRISPR-Cas9 yielded two distinct mutants: one carrying a missense mutation at amino acid (aa) position 73 (methionine to leucine substitution) and another harboring a frameshift mutation between aa positions 50 and 142. Flow cytometric analysis (FACS) of T_1_ seedlings from the *ZmPOD65*^+/Met→Leu^ line and T_2_ seedlings from the *ZmPOD65*^+/frameshift^ line revealed HIRs of 7.7% and approximately 1%, respectively. In contrast, no haploid seedlings were detected among mutants of *ZmPOD60–1* or *ZmPOD60-2*, underscoring a specific role for *ZmPOD65* in haploid induction and supporting the critical involvement of ROS dynamics in this process.

Based on these findings, Jiang et al. (2022) proposed a mechanistic model wherein a centrally triggered ROS burst underpins haploid induction during the second mitosis of pollen development: (1) elevated phosphatidylcholine (PC) levels in sperm cells stimulate excessive ROS production; (2) this oxidative stress disrupts the redox homeostasis of the metabolome, leading to sperm DNA damage and initiating multilayered regulatory responses; (3) persistent DNA breakage, extending from centromeric regions to the entire sperm genome, surpasses the capacity of DNA repair mechanisms. Following fertilization, the fragmented male genome is progressively degraded, ultimately resulting in haploid progeny.

### 2.3 Genes involved in maternal and paternal haploid induction

#### 2.3.1 *CENH3* gene

*Centromere-specific histone H3 (CENH3)* is highly conserved of all known kinetochore proteins (Henikoff et al., 2001). Data from several organisms indicate that *CENH3* replaces histone H3 on active centromeric DNA (Yoda et al., 2000; Ahmad and Henikoff, 2001; Lo et al., 2001; Blower et al., 2002) and is required to recruit other key kinetochore proteins such as CENP-C (Hooser et al., 2001; Ando et al., 2002). Each of the known *CENH3s* shares a common histone H3 core sequence, but they diverge in the N-terminal tail and an internal region known as loop 1 (Talbert et al., 2002). The histone fold domain (HFD) of *CENH3* comprises six secondary structural elements, including four alpha helices (αN, α1, α2, and α3) and two loops (Feng et al., 2020). Both diverged regions interact with DNA in the nucleosome and show evidence of adaptive evolution, suggesting that *CENH3* serves as a linker molecule between the rapidly evolving centromeric DNA and the conserved kinetochore machinery (Malik and Henikoff, 2002).

Because of its close association with DNA in the context of the centromeric nucleosomes, *CENH3* has been used as a tool to identify the centromere sequences that interact with the kinetochore. *In situ* hybridization (Haaf and Ward, 1994; Sart et al., 1997) and chromatin immunoprecipitation (ChIP) have been used to show that maize *CENH3* is present in kinetochores throughout the cell cycle. ChIP analysis indicates that maize *CENH3* interacts strongly with the centromeric satellite CentC but does not interact with noncentromeric DNA sequences. The ChIP assays also demonstrate that a recently isolated centromeric retrotransposon in maize (CRM) interacts with *CENH3* throughout its length. These data provide strong support for the idea that specific sequences confer centromere identity in maize and that a conserved retrotransposable element is part of the functional centromere (Zhong et al., 2002).

Ravi and Chan (2010) studied on genome elimination of the centromere-specific histone *CENH3* gene in *Arabidopsis thaliana* plants by fluorescent protein (GFP)−tailswap. The result found that haploids are efficiently generated at 25–45% from a cross of GFP–tailswap with wild-type. However, several unusual phenotypes in the F_1_ progeny were observed, such as approximately 80–95% of fertilized ovules aborted early in development, leading to inviable seeds.

Later, Maheshwari et al. (2015) investigated the function of the *CENH3* gene in maize by genetic modification under the control of the endogenous *CENH3* promoter. *Cenh3-1/CENH3* heterozygotes were created using these transgenes. In the T_1_ generation, they discovered *Zea mays CENH3* transformants in the homozygous background *cenh3–1* are embryo lethality. This finding demonstrates that *CENH3* may play a significant role in haploid induction in these plant groups (Rai et al., 2023).

Then Kelliher et al. (2016) engineered two haploid inducer lines, including *CENH3*−/− and *CENH3*: RNA interference (RNAi) lines by AcGREEN-tailswap-*CENH3*. Subsequently, those inducers were test crosses to wild-type plants and evaluated HIRs. Results found that *CENH3*: RNAi lines did not consistently knock down *CENH3* and only occasionally produced haploids. When backcrossed as males, the *CENH3*−/− showed HIR of gynogenic in many hemizygous individuals reached a maximum of 3.6%, with a lower rate of male sterility. These findings suggest that *in vivo* haploid induction systems may be engineered into maize plants using *CENH3*-tailswap transgenes. However, the use of a single RNAi copy line doesn’t prove effective for haploid production, indicating the need for a multi-copy RNAi line (Rai et al., 2023).

After that Wang et al. (2021) presented a method for producing haploid progeny involving mating maize heterozygous lines with wild-type plants in both directions (*cenh3* null mutants +/*cenh3* (*gl1, gl8*, and *cenh3*-mu1015598 transposon insertion lines). When +/*cenh3* heterozygotes were crossed as males, 0.5% of the progeny were glossy, but 5.2% of the offspring were glossy when +/*cenh3* plants were used as females. genome elimination (GE) in the gametophyte was detected by the genotype for *CENH3*, proving that *CENH3* dilution during postmeiotic cell divisions before gamete production is what causes centromere failure.

Recently Meng et al. (2022) developed maternal haploid inducer lines derived from Stock6 (3.2%) with genetic modifications in *CENH3* gene. Subsequently, overexpressed fluorescently protein-tagged maize *CENH3* to modify the Stock6-derived inducer lines (CAUHOI and CAU5), resulting in an increased maternal HIR of 6.1%. The HIR increased to 16.3% when replacing the full-length *CENH3* in the tagged expression cassette with a tail-altered version in CAU5^M-tailswap-RFP^, while it increased to 8.8% from 5.3% in CAUHOl^M-tailswap-RFP^. These findings provide a potentially workable hypothesis for further improving the induction rates of maize CAU5 (*mtl/zmpla1/nld + zmdmp*) and CAUHOI (*mtl/zmpla1/nld*) inducer lines (maternal haploid) and indicate the potential of combining two *in-vivo* haploid induction strategies to improve the success rate of generating haploid populations in maize (Rai et al., 2023).

Most recently, it was found that *CENH3* not only has broader applicability as both maternal and paternal parents in haploid induction but also exhibits temperature sensitivity due to reduced HIR when the temperature decreases. As the previous report indicated that *CENH3* disruption caused embryo lethality (Maheshwari et al., 2015). Yang et al. (2025) proposed moderate modifications of *CENH3* using microRNA-induced gene silencing (MIGS) driven by the RPS5a promoter (Felippes et al., 2012), which is activated only during gametogenesis and early embryogenesis (Weijers et al., 2001) to knock down the gene in *Arabidopsis thaliana*. The result found that at 22°C, a transgenic line (pRPS5a::MIGS #1) displayed a low HIR of 0.2%, while the HIR increased significantly to 0.2%–3.4% at 25°C and 6.1%–14.2% at 30°C across all lines, and aborted seed rates also rose with temperature. Also found that the combination of null *cenh3–*1 mutant with pRPS5a::MIGS and GFP-*CENH3* in heterozygous form can increase the HIR by 1.31-fold to 1.97-fold compared with that of the null *cenh3–1* mutant with GFP-*CENH3* alone, and increase to 66.7% and 61.4% in T_2_ and T_3_ generations in homozygous form at 22°C. And found that all the plants were maintaining healthy, fertility, and pollen viability.

## 3 Genetic identification of haploid genome

### 3.1 *R1‐navajo* gene

In Nanda and Chase 1966, observed *R1-navajo* (*R1-nj*) phenotypic causes the purple color in the aleurone layer on the crown region of the endosperm and scutellum of the embryo. Subsequently, Geiger (2009) suggested that the *R1-nj* expression requires the presence of functional *A1, A2, Bz1, Bz2, C1, C2*, and *Pr1* genes involved in the anthocyanin biosynthesis pathway. Which, anthocyanin accumulation in aleurone requires the joint action of *R1* (*red color1*) and *C1* (*colored aleurone1*) transcription factors (Chandler et al., 1989), the *R1* gene encode bHLH transcription factors (Ludwig et al., 1990; Goff et al., 1992), while *C1* encode MYB-homologous DNA binding domain proteins (Cone et al., 1993a). The *R1* gene present on chromosome 10 governing anthocyanin pigmentation on maize kernels, has been widely used as a color marker for haploid identification (Melchinger et al., 2015) due to its easy and quick identification of haploid kernels at the seed stage during *in vivo* haploid induction process in maize (Chaikam et al., 2015).

However, Chaikam et al. (2016) reported that *R1-nj* fails in haploid identification when (i) *R1-nj* expression is completely inhibited by dominant color inhibitor genes; (ii) *R1-nj* expression is segregating among the kernels of the source germplasm; and (iii) *R1-nj* marker expression is poor for anthocyanin intensity or the marked area. In a set of 897 tropical inbred lines, complete inhibition of *R1-nj* was shown to frequently occur (∼30%) and was attributed to the presence of the color inhibitor *C1-I* (*c1*) (Chaikam et al., 2015). In addition, ∼70% of the 155 landraces and ∼40% of 157 breeding populations showed segregation for *R1-nj* expression. Hence, the use of *R1-nj* is not efficient in such germplasm (Chaikam et al., 2015). Even when expressed, poor intensity of the *R1-nj* marker expression can result in high rates of misclassification in temperate flint germplasm (Röber et al., 2005; Melchinger et al., 2014) and tropical landraces (Prigge et al., 2011). Physiological factors such as high moisture content (Rotarenco et al., 2010) and the development of air pockets underneath the pericarp (Prigge et al., 2011) can also affect the efficiency and accuracy of *R1-nj* based haploid identification. Another potential problem is masking the *R1-nj* phenotype by natural anthocyanin coloration in the seed, especially in the pericarp, of maize landraces.

### 3.2 *Pl-1* gene

Anthocyanin accumulation in maize tissues requires the expression of at least 20 loci, comprising genes encoding biosynthetic enzymes and two groups of regulatory genes (basic helix-loop-helix; bHLH and MYB-related transcription factors) responsible for the developmental and tissue-specific pigmentation (Mol et al., 1998). *Pl1* genes located on chromosome 6 encode R2R3 MYB transcription factors regulating anthocyanin biosynthesis (Cone et al., 1993b; Paz-Ares et al., 1986). *Pl1* controls pigmentation in the root seedlings, in adult vegetative tissues, and in the pericarp, a maternally derived seed integument (McCarty et al., 1989). Therefore, Rotarenco et al. (2010) employed both the *B1* and *Pl1* marker genes to develop Procera Haploid Inducer (PHI) to allow haploids to be discriminated by the lack of red expression in maize seedling stems and roots.

Dermail et al. (2024) reported that it is imperative to evaluate the natural seedling root pigmentation of vast maize source germplasm in a targeted breeding program before implementing this marker. Germinating abundant induced seeds is also resource-intensive. Since the *Pl1* gene is light-dependent (Emerson, 1921; Briggs, 1966; Coe et al., 1988), the seedling roots of some true haploids can turn red when exposed to light, making the *Pl1*-based haploid selection prone to increased false negatives (Trentin et al., 2020). To prevent the roots from light exposure, it is suggested that enough soil be provided to cover the seedlings and to keep the roots under the plug trays (Vanous et al., 2017). In another case, the female parent was used with the *pl1* allele.

### 3.3 *C1-I* gene

**Figure 1:**
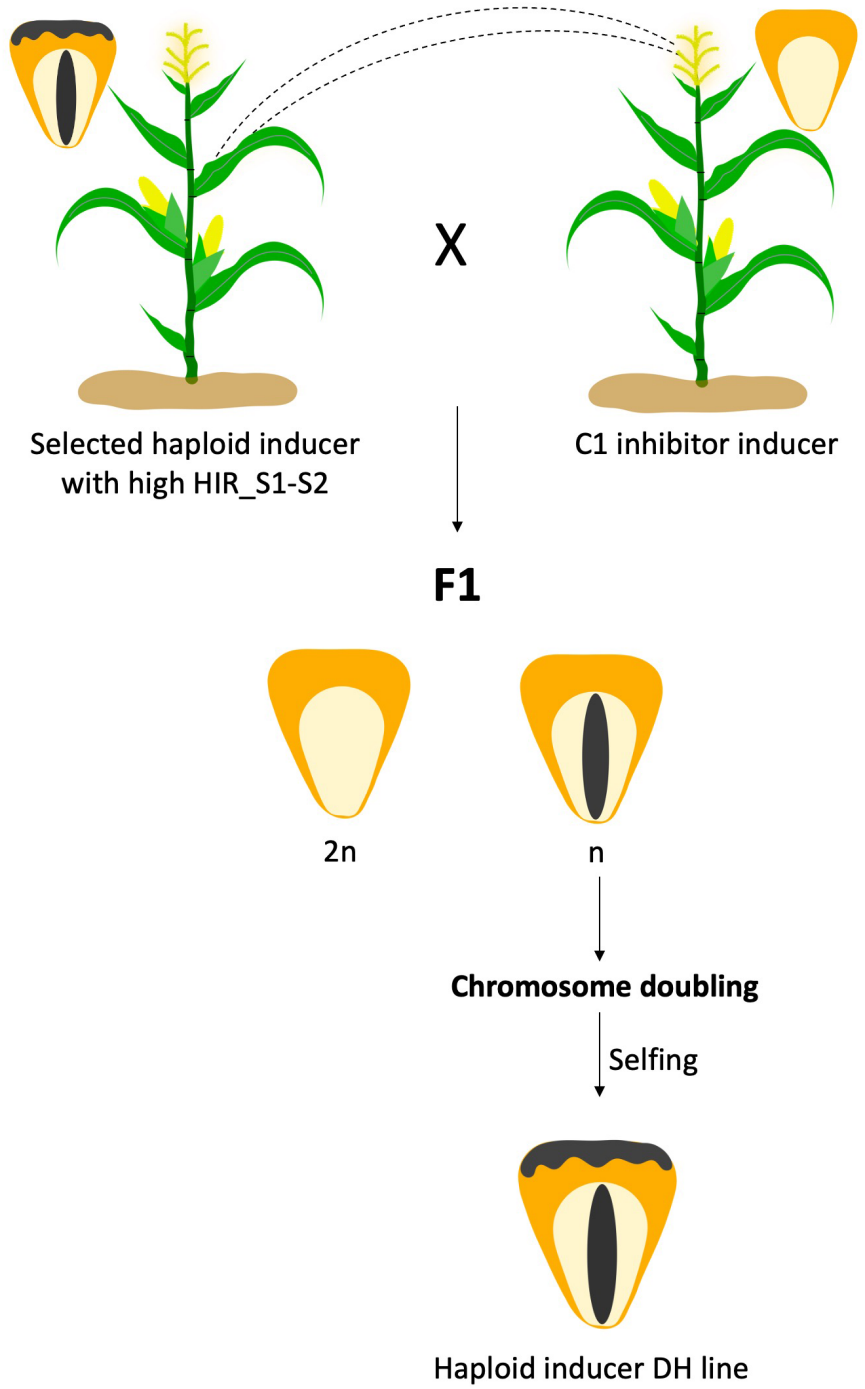
*C1-Inhibitor* (*C1-I*) haploid inducer development system.

The presence of *C1-Inhibitor* (*C1-I*), a dominant mutant of *C1* gene present on chromosome 9 in the genetic background, has been identified as the key factor that interferes with the expression of kernel color (Coe, 1962). Through sequence analysis, Chaikam et al. (2015) reported the presence of the 8 bp InDel and a SNP (A–G transition) in exon-3 that differentiated the *C1* from *C1-I* allele. The *C1-I* gene is avoided in *R1-nj* selection system. In haploid inducer development, *C1-I* becomes a favorable allele for Inbred haploid inducer development. In Chen et al., 2024, reported *C1-I* inducer can be used as a male pollinate to normal *R1-nj* haploid inducer females, producing haploid seeds (n) with only anthocyanin pigmentation at the embryo but no pigment in the endosperm. While no pigmentation is produced in diploid seeds (2n) (**Figure 1**). It was found that the HIR of *C1-I* inducers ranged between 5.8% and 12.0%. Overall, the success rate of DH production was 13% on average across the 23 different inducer crosses.

### 3.4 Kernel oil content

Maize is a valuable resource of vegetable oil for human consumption, although it is primarily grown for animal feed. Maize oil mainly accumulates in the embryo, and it is typically comprised of approximately 11% palmitic acid (C16:0), 2% stearic acid (C18:0), 24% oleic acid (C18:1), 62% linoleic acid (C18:2), and 1% linolenic acid (C18:3) (Lambert, 2001). Since maize kernel oil concentration became useful for animal feeding, the Illinois Long-Term Selection Experiment was initiated in 1896 to investigate changes in maize kernel chemical composition resulting from the selection for protein and oil concentration (Hopkins, 1899).

In Brown 1971, suggested that the allelic variation at six isozyme loci among the strains Illinois High Oil (IHO) and Illinois Low Oil (ILO) after 68 cycles of selection in the Illinois Long Term Selection Experiment could be largely accounted for by random genetic drift. Significant linear allozyme frequency changes at eight loci were associated with 25 selection cycles for increased oil concentration in the Alexho maize synthetic (Kahler, 1985), suggesting the possible presence of genes controlling oil concentration at or near these allozyme loci. In Sughroue and Rocheford 1994, identified 49 RFLP probes distributed throughout the maize genome whose changes in frequency among oil strains in the Illinois Long Term Selection Experiment are consistent with the selection response. In the same year, Goldman et al. (1994) conducted to determine the number and magnitude of quantitative trait loci (QTL) influencing kernel oil concentration and kernel weight in a maize population derived from a cross of Illinois High Protein (IHP) × Illinois Low Protein (ILP). One hundred polymorphic RFLP loci spaced throughout the maize genome were scored in a segregating population of 100 S_1_ families. The result found that 25 marker loci significant (P < 0.05 level) QTL associations were detected on Chromosome Arms 2L, 4L, 6L, and 8L with oil concentration.

Since the mechanism is still a mystery, this topic has become popular for decades. Many QTL were studied and harvested from different research groups, including Mangolin et al. (2004), were generated a genetic map with 75 microsatellites, and the thirteen QTLs were mapped on eight chromosomes and explained 26.64% of the genetic variation. Willmot et al. (2006) reported that QTLs of KOC were detected in chromosomal bins 2.09, 3.04,6.04, 8.04, 8.05, and 8.07. Wassom et al. (2008) generated a genetic map with a length of 1486 cM from 110 markers. Multiple regression models with QTL detected by composite interval mapping (CIM) explained 46.9% of phenotypic variance for oil in BC_1_S_1_s. A 22 cM-interval on chromosome 6 explains about 36.7% of the BC_1_S_1_ phenotypic variation for KOC. In the same year, Zheng et al. (2008) reported a high-oil QTL (qHO6) on chromosome 6 mapped from BC_3_S_2_ population of PH09B (3.2) with ASKC28IB1 (18.1%), affecting maize seed oil and oleic acid contents encodes an acyl-CoA: *diacylglycerol acyltransferase* (*DGAT1-2*), which catalyzes the final step of oil synthesis. Found that the phenylalanine insertion in diacylglycerol acyltransferase (*DGAT1-2*) at position 469 (F469) led to increased oil and oleic acid contents, and the *DGAT1–2* allele increased oil and oleic acid contents up to 41% and 107%, respectively.

The new approach of KOC is not only for consumption but to be used as maize inbred lines development through haploid inducers. As Li et al. (2009) applied high-oil traits to haploid inducers. The Stock-6-derived, haploid-inducing line CAUHOI with high KOC (78.05 g/kg) was used as the pollinator to produce maternal haploids from the maize hybrid ZD958 with low KOC (35.42 g/kg). The result showed all the normal haploids had low KOC with a mean value of 33.25 g/kg, which was a little lower than that of the selfed kernels of ZD958. Among the 277 diploid-like haploids, most kernels had a low KOC similar to that of normal haploids. As a whole, the average KOC of all the haploids, including the diploid-like haploids, was 37.50 g/kg, significantly lower than the average KOC (60.03 g/kg) of hybrid kernels.

Due to the advances in technology supported the study was conducted be deeply and more specific, and various genes related to KOC were reported, including Shen et al. (2010) reported that overexpression of two genes in maize, *LEAFY COTYLEDON1* (*ZmLEC1*) increases seed oil to 48% but reduces seed germination and leaf growth in maize. An overexpression of *ZmWRI1* results in an oil increase similar to overexpression of *ZmLEC1* without affecting germination, seedling growth, or grain yield. These results highlight *ZmWRI1* as a promising target for increasing oil production in crops. Li et al. (2011) reported QTL-Pal9 was mapped to a 90-kb region, in which we identified a candidate gene, *fatb* (*Zmfatb*), which encodes acyl-ACP thioesterase. An 11-bp insertion in the last exon of *Zmfatb* decreases palmitic acid content and concentration, leading to an optimization of the ratio of saturated to unsaturated fatty acids while not affecting total oil content. Han et al. (2016) found that *ZmSAD1*, supported by both the QTL and an expression QTL, had the largest effect on C18:0/C18:1. One nonsynonymous single-nucleotide polymorphism in exon 3 and one 5-bp insertion/deletion in the 3′ untranslated region was further shown to contribute to the natural variation in C18:0/C18:1 according to *ZmSAD1*-based association mapping. After that, Fang et al. (2021) reported two important candidate genes, *GRMZM2G101515* and *GRMZM2G022558*, which were further verified to be associated with C20:0/C22:0 and C18:0/C20:0, respectively, according to a gene-based association analysis. The first gene encodes a kinase-related protein with unknown function, while the second gene encodes *fatty acid elongase 2* (*fae2*) and directly participates in the biosynthesis of very long-chain fatty acids in Arabidopsis.

Over 30 years of QTL mapping in KOC revealed many QTLs and genes. However, the mechanisms are still less understood. Melchinger et al. (2013) reported that double fertilization with pollen from high-oil inducers resulted in viable diploid seeds showing improved KOC, while haploid seeds due to either single fertilization or post-embryo male genome elimination have low KOC and or similar KOC as source germplasm. Four factors determine the effectiveness of KOC for haploid selection, as follows: (1) the HIR of the inducer lines; (2) the KOC difference between haploid and diploid fractions; (3) the phenotypic variance of KOC within seed fractions; and (4) the optimum thresholds of KOC values for haploid and diploid determinations (Dermail et al., 2024).

## 4 Genetic related to genome doubling in maize

Spontaneous haploid genome doubling (SHGD) plays a critical role in maize genome doubling because it requires zero inducing methodology, but the genetic mechanism to recover pollen viability itself. It was first reported by Chase (1947, 1949), who observed the occurrence of diploid tissue sectors arising in occasional cells, particularly within the anthers and ovules. These early findings provided foundational insights into spontaneous diploidization processes in maize. Nearly five decades later, renewed interest in the mechanisms and genetic basis of SHGD has led to significant progress in dissecting its underlying biology. Testillano et al. (2004) studied SHGD during early stages of *in vitro* maize microspore embryogenesis. The result found that nuclear fusion occurred at the 5-to-7-day developmental stage within the embryonic domain, potentially leading to polyploidy in microspore-derived endosperm tissues.

Further expanding on the origins of SHGD, Wu et al. (2014) suggested two distinct pathways: (i) maternal plants producing 2n female gametes through abnormal meiosis, followed by parthenogenesis development into diploids, and (ii) formation of haploid zygotes through normal meiosis and induction, followed by genome doubling via abnormal mitosis during seed development. The detection of mixoploid individuals among DH_0_ plants—with fewer than 10% haploid cells—supports the latter pathway, suggesting incomplete genome doubling in initially haploid zygotes.

Ren et al. (2017) mapped QTL for haploid male fertility (HMF) and identified three loci—*qhmf1*, *qhmf2*, and *qhmf4* —in haploid populations derived from ‘4F1’ and ‘Yu87-1/Zheng58’. Thirteen polymorphic markers were developed to saturate the *qhmf4* region, narrowing it down to ~800 kb interval flanked by markers IND166 and IND1668. Notably, the absence of *first division* (*afd1*) gene within this interval emerged as a candidate influencing HMF.

Ma et al. (2018) identified genes associated with HMF through genome-wide association study (GWAS) using a diversity panel of 481 maize inbred lines crossed with ‘Mo17’ and ‘Zheng58’. Three candidate genes were identified — *GRMZM2G469593, GRMZM2G174092* (both on bin 2.05), and *GRMZM2G056236*, the latter being annotated as involved in sexual reproduction, potentially mediating HMF restoration.

Molenaar et al. (2019) investigated the genetic architecture of SHGD using modified quantitative-genetic models across haploid progeny derived from ten DH lines and corresponding diallel crosses. Recurrent selection over three breeding cycles revealed predominant additive genetic effects, although epistasis was also significant. Entry-mean heritability for SHGD exceeded 0.91, while single-plant heritability ranged between 0.11 and 0.19, indicating that SHGD is a polygenic trait. Subsequently, Chaikam et al. (2019b) identified genes influencing spontaneous fertility in maize haploids. The 214,520 markers from the GBS platform were used for GWAS analyses. The result found that putative candidate genes identified on chromosomes 1, 3, 4, 5, and 10 for HMF; one of the genes named *GRMZM2G478417* is annotated as being involved in pollen mother cell meiosis. Another SNP (S4_223079313) present within the *GRMZM2G041530* gene is engaged in *GDSL-like lipase/acylhydrolase* activity, which has a role in seed development.

Ren et al. (2020) identified major QTL and a gene associated with SHGD in two haploid populations derived from inbred lines A427 (SHGD rate: 0.65), GF3 (0.29), and Wf9 (0), which displayed contrasting SHGD efficiencies. Three QTL—*qshgd1* (Chr 5), *qshgd2* (Chr 6), and *qshgd3* (Chr 9)—were mapped. To pinpoint candidate genes within the *qshgd1* region, RNA-Seq analysis was conducted comparing GF1 and GF5. A form *in-like protein 5* gene was differentially expressed within this region, suggesting a potential role in cell division processes relevant to SHGD.

Subsequently, Trampe et al. (2020) reported a major QTL on chromosome 5 that accounted for over 45% of the phenotypic variance in haploid male fertility (HMF) across multiple environments. A biparental mapping population of 220 F_2:3_ families was developed from a cross between A427 (high HMF) and CR1Ht (moderate HMF). Genotyping was performed using a high-density linkage map containing 4,171 SNP markers distributed across all 10 chromosomes, with an average inter-marker distance of 0.51 cM. The major QTL was localized near the centromere of chromosome 5, spanning the 101.6-104.8 Mb interval, based on the ZmB73v4 genome assembly (Ren et al., 2020).

Verzegnazzi et al. (2021) evaluated the impact of the A427 genotype using doubled haploid (DH) and single seed descent (SSD) lines derived from BS39 and BS39 × A427 crosses. Case-control association mapping of 663 inbred lines from four population sets identified a locus associated with SHGD near the centromere of chromosome 5, consistent with findings from Ren et al. (2020) and Trampe et al. (2020). Haplotype-sharing analysis showed an almost exclusive contribution of the A427 genomic region to the BS39 × A427_DH lines on chromosome 5, implicating a key allele in this region as a determinant of SHGD.

More recently, Foster et al. (2024) further refined the candidate gene region within qshgd1 on chromosome 5. A set of 232 recombinant BC_1_F_1_ individuals was developed using A427 (high HMF) as the donor and Wf9 as the recurrent parent. A linkage map constructed using 17 SNP markers spanned approximately 45 Mb in physical distance and 2.4 cM in genetic distance. A large non-recombining region, from marker 11 (96.01 Mb) to marker 14 (119.77 Mb), encompassed the centromeric and pericentromeric domains. Within this “recombination dead zone,” sequence alignment and gene model filtering identified 79 potential candidate genes. From these, 10 genes—*Zm00001eb234380, Zm00001eb234410, Zm00001eb234730, Zm00001eb234840, Zm00001eb234920, Zm00001eb235200, Zm00001eb235320, Zm00001eb235400, Zm00001eb235450*, and *Zm00001eb235700*— were prioritized based on high expression in mitotically and meiotically active plant tissues.

Despite these advancements, the underlying molecular and cellular mechanisms governing SHGD remain incompletely understood. Future research integrating high-resolution genomic mapping, functional validation, and cytological characterization will be critical to fully elucidate the genetic architecture and biological basis of this agriculturally valuable trait.

## 5 Genetically related to haploid induction applied to other crops

Haploid induction has been successfully applied in the Maize breeding program. Therefore, this trait advancement extended to other crops such as rice, the knockout mutations of *OsMATL* led to an increased HIR up to 2-6% (Yao et al., 2018) and Wang et al. (2022) found that asynchronous heading dates between haploid inducers and acceptors became limited for this technology, therefore diverse heading dates haploid inducers with *MTL* gene mutation by CRISPR-Cas9 system were generated and shown HIR of 2.8–12.0%. In wheat, Liu et al. (2020) found that *MTL/ZmPLA1/NLD* homologues with three *TaPLA* genes, in self-pollinated progenies of all knockout lines, showed average HIR ranged from 5.88% to 15.66%. Subsequently, Lv et al. (2020) developed genome-edited *TaCENH3α-heteroallelic* combinations, which are paternal inducer lines in wheat with HIR of approximately 7%. *DMP* gene has become a model haploid induction gene in dicot plants because it is conserved in both monocots and dicots. In tomato, Zhong et al. (2022a) generated a maternal haploid inducer with *sldmp* gene mutation using a CRISPR-Cas9 mutagenesis construct that includes the FAST-Red marker. After crossing 36 different female genotypes with the *sldmp* inducer lines, HIR increased in the range of 0.5-3.7%. In *Brassica napus* and Nicotiana tabacum, Zhong et al. (2022b) edited *DMP* gene using the CRISPR-Cas9 system. The result found that average amphihaploid induction rates of 2.4% and 1.2% in *Brassica napus* and *Nicotiana tabacum*, respectively. In *legume*, Wang et al. (2022) found that *ZmDMP* were similar to *MtDMP8* (*Medtr7g010890*) and *MtDMP9* (*Medtr5g044580*) genes with 63.9% and 62.8% sequence identity, respectively, and found HIR between 0.29% to 0.82% among the T_2_ progeny of *mtdmp8 mtdmp9* mutant lines. In watermelon, Chen et al. (2023) found that *ClDMP3* mutant lines can increase the HIR up to 1.12%. Not only *MTL/ZmPLA1/NLD* and *DMP*, but *CENH3* is also widely used as a haploid induction gene in vegetable crops, such as onion. Manape et al. (2024) downregulated *AcCENH3* using the RNAi approach without complementation in five independent lines in onion led to HIR showing up between 0 and 4.63%. And in the *Brassica* crop, Han et al. (2024) advanced a *BolC8t52879H* (*BoCENH3*)-based haploid induction to generate paternal CMS broccoli haploid inducer lines with HIR of 0.50-1.29%.

## 6 Challenges and research outlook

The advent of haploid induction and doubled haploid DH technologies has markedly accelerated the pace of maize breeding, yet critical barriers persist. Although substantial progress has been made in identifying key genetic determinants such as *IG1, ZmPLA1/MTL/NLD, ZmDMP, ZmPLD3, ZmPOD65*, and *CENH3*, the intricate molecular circuitry governing haploid induction, genome elimination, and spontaneous haploid genome doubling (SHGD) remains incompletely understood. Genotype specificity, environmental modulation of phenotypic markers, and dependence on cytotoxic chromosome-doubling agents constrain the scalability and universality of current protocols.

Future research must pivot toward elucidating the oxidative and DNA damage signaling networks central to haploid induction, while pioneering molecular marker systems that transcend the limitations of anthocyanin-based identification. Advances in genome editing—particularly CRISPR-mediated reprogramming of haploid induction loci—hold the promise of engineering next-generation inducers with superior efficiency, stability, and adaptability across diverse genetic backgrounds. Furthermore, leveraging SHGD through genomic prediction and selection frameworks could substantially reduce reliance on chemical treatments, offering a more sustainable path forward.

Ultimately, the fusion of functional genomics, precision genome engineering, and predictive breeding algorithms heralds a transformative shift toward precision doubled haploid breeding—a paradigm poised to accelerate genetic gain, enhance crop resilience, and contribute meaningfully to global food security in the face of mounting environmental and demographic pressures.
